# Supplementary material for: Protective effects of dapagliflozin against oxidative stress-induced cell injury in human proximal tubular cells
Source: PLoS One. 2021 Feb 19;16(2):e0247234. doi: 10.1371/journal.pone.0247234 (PMC7894948; doi:10.1371/journal.pone.0247234)
Supplement: S1 Fig — (PPTX) [file pone.0247234.s001.pptx]

## Slide 1
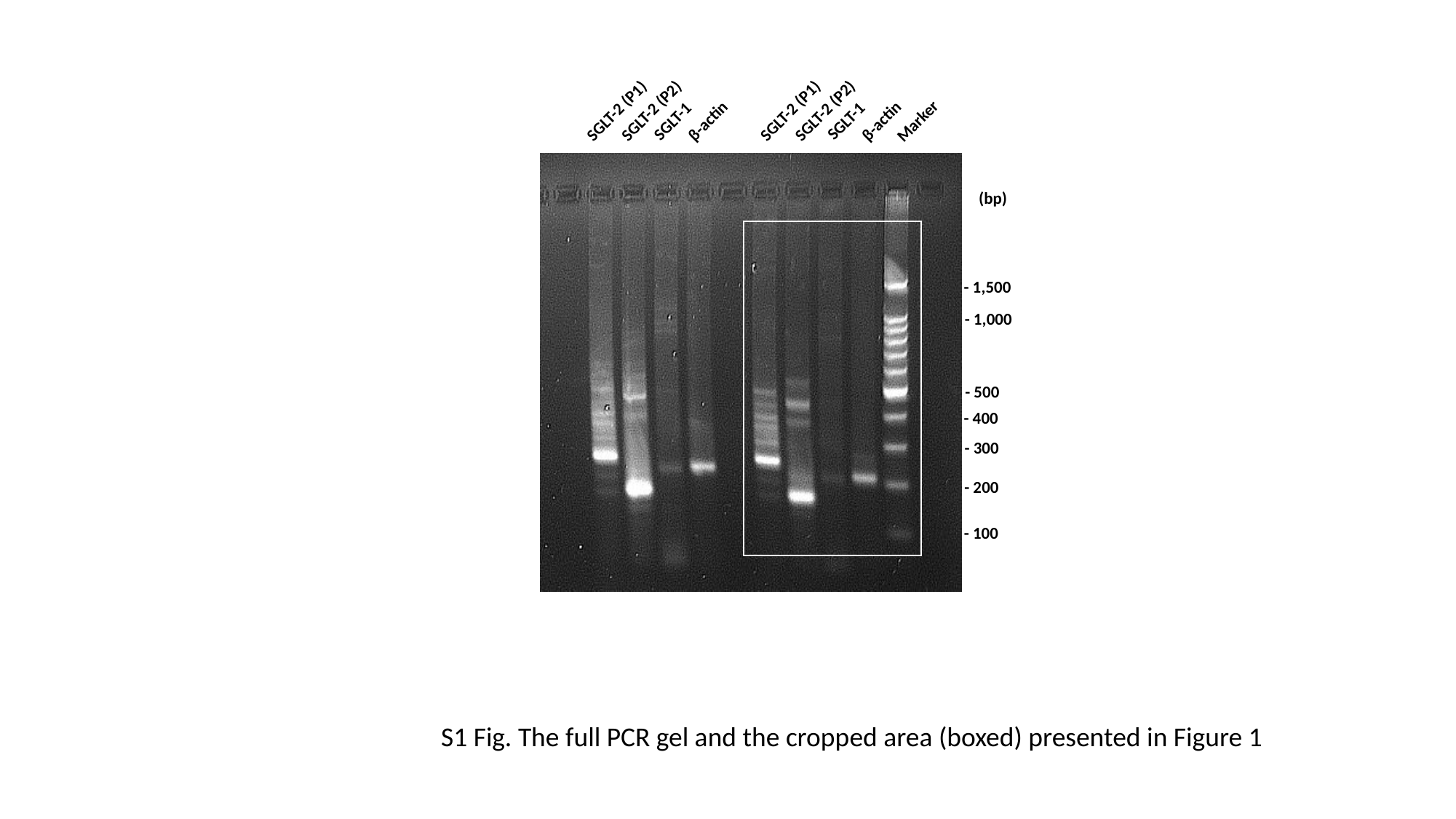

SGLT-1
SGLT-1
SGLT-2 (P2)
SGLT-2 (P2)
SGLT-2 (P1)
SGLT-2 (P1)
β-actin
β-actin
Marker
(bp)
- 1,500
- 1,000
- 500
- 400
- 300
- 200
- 100
S1 Fig. The full PCR gel and the cropped area (boxed) presented in Figure 1
