# Supplementary material for: Protective effects of dapagliflozin against oxidative stress-induced cell injury in human proximal tubular cells
Source: PLoS One. 2021 Feb 19;16(2):e0247234. doi: 10.1371/journal.pone.0247234 (PMC7894948; doi:10.1371/journal.pone.0247234)
Supplement: Attachment — Submitted filename: Response to reviewers.pdf [file pone.0247234.s002.pdf]

Dear Dr R Franco,

Thank you for your careful editing for the manuscript.

We have completed the revision and carefully proofread the whole paper. One postdoc in my lab also helped us to do proofreading.

In addition, the manuscript has been reformatted according to the PLOS instruction. The full PCR gel image is listed as Supporting Information. We hope this version is acceptable.

The response to reviewers is given below.

Yours

Sam Xu (Shang-Zhong Xu)

===
Dr S.Z. Xu, PhD, MD
Reader in Diabetes, Endocrinology & Metabolism
Hull York Medical School
102 Allam Building, Cottingham Road, Hull, UK, HU6 7RX
https://www.hyms.ac.uk/about/people/sam-xu

## Response to editor:

1. Please ensure that your manuscript meets PLOS ONE's style requirements, including those for file naming. The PLOS ONE style templates can be found at
https://journals.plos.org/plosone/s/file?id=wjVg/PLOSOne_formatting_sample_main_body.pdf and
https://journals.plos.org/plosone/s/file?id=ba62/PLOSOne_formatting_sample_title_authors_affiliations.pdf

The manuscript has been reformatted as PLOS ONE.

2. Please provide additional information about each of the cell lines used in this work, including the source of the inducible TRPM2 cells and any quality control testing procedures conducted (authentication, characterisation, and mycoplasma testing). For more information, please see http://journals.plos.org/plosone/s/submission-guidelines#loc-cell-lines.

The cell line resource and related references are added.

3. At this time, we ask that you please provide scale bars on the microscopy images presented in Figure 2E and 3C and refer to the scale bar in the corresponding Figure legend.

Added.

4.We note that you have indicated that data from this study are available upon request. PLOS only allows data to be available upon request if there are legal or ethical restrictions on sharing data publicly. For more information on unacceptable data access restrictions, please see http://journals.plos.org/plosone/s/data-availability#loc-unacceptable-data-access-restrictions.
In your revised cover letter, please address the following prompts:
a) If there are ethical or legal restrictions on sharing a de-identified data set, please explain them in detail (e.g., data contain potentially sensitive information, data are owned by a third-party organization, etc.) and who has imposed them (e.g., an ethics committee). Please also provide contact information for a data access committee, ethics committee, or other institutional body to which data requests may be sent.

No ethical issue for the paper.

b) If there are no restrictions, please upload the minimal anonymized data set necessary to replicate your study findings as either Supporting Information files or to a stable, public repository and provide us with the relevant URLs, DOIs, or accession numbers. For a list of acceptable repositories, please see http://journals.plos.org/plosone/s/data-availability#loc-recommended-repositories.

We have given all the data in the manuscript and gel image in the supporting information. So we can give the statement “All relevant data are within the manuscript and its Supporting Information files”. In addition, the experimental procedure has also been given for public access: https://www.protocols.io/view/protocol-for-intracellular-ca2-detection-using-fle-bnubmesn

5.PLOS ONE now requires that authors provide the original uncropped and unadjusted images underlying all blot or gel results reported in a submission’s figures or Supporting Information files. This policy and the journal’s other requirements for blot/gel reporting and figure preparation are described in detail at https://journals.plos.org/plosone/s/figures#loc-blot-and-gel-reporting-requirements and https://journals.plos.org/plosone/s/figures#loc-preparing-figures-from-image-files. When you submit your revised manuscript, please ensure that your figures adhere fully to these guidelines and provide the original underlying images for all blot or gel data reported in your submission. See the following link for instructions on providing the original image data: https://journals.plos.org/plosone/s/figures#loc-original-images-for-blots-and-gels.

Full length gel image was shown as supplementary figure.

6. Please ensure that you refer to Figure 8 in your text as, if accepted, production will need this reference to link the reader to the figure.

Thank you. Now corrected.

7.We suggest you thoroughly copyedit your manuscript for language usage, spelling, and grammar. If you do not know anyone who can help you do this, you may wish to consider employing a professional scientific editing service.

We have carefully proofread the paper. My colleague helped to proofread as well. The version with track changes is uploaded for reference.

## Response to reviewer:

1. Is the manuscript technically sound, and do the data support the conclusions?
The manuscript must describe a technically sound piece of scientific research with data that supports the conclusions. Experiments must have been conducted rigorously, with appropriate controls, replication, and sample sizes. The conclusions must be drawn appropriately based on the data presented.
Reviewer #1: Yes

Thank you.

2. Has the statistical analysis been performed appropriately and rigorously?
Reviewer #1: No

The statistical methods and n number are clearly given in the text or figure legends. Paired *t* test was used for the results before and after treatment, and unpaired *t* test for two group comparison. ANOVA with Bonferroni's post-hoc analysis was used where appropriate.

3. Have the authors made all data underlying the findings in their manuscript fully available?
The PLOS Data policy requires authors to make all data underlying the findings described in their manuscript fully available without restriction, with rare exception (please refer to the Data Availability Statement in the manuscript PDF file). The data should be provided as part of the manuscript or its supporting information, or deposited to a public repository. For example, in addition to summary statistics, the data points behind means, medians and variance measures should be available. If there are restrictions on publicly sharing data—e.g. participant privacy or use of data from a third party—those must be specified.
Reviewer #1: No

Data are available. See response to editor.

4. Is the manuscript presented in an intelligible fashion and written in standard English?

PLOS ONE does not copyedit accepted manuscripts, so the language in submitted articles must be clear, correct, and unambiguous. Any typographical or grammatical errors should be corrected at revision, so please note any specific errors here.
Reviewer #1: No

We have carefully edited the manuscript and done proofreading.

5. Review Comments to the Author
Please use the space provided to explain your answers to the questions above. You may also include additional comments for the author, including concerns about dual publication, research ethics, or publication ethics. (Please upload your review as an attachment if it exceeds 20,000 characters)
Reviewer #1: Question 1: In the section of the results, the subtitles should highlight the central idea of the paragraph. Therefore, the results section should be elaborated in more detail.

We have reworded some subtitles and changed some description in the text.

Question 2: How did the authors choose to study on SGLT1 and SGLT2?

Unclear for this question. The SGLT2 is the target for dapagliflozin and many beneficial effects of the compound including cardiovascular and renal protection have been reported; however, it is unclear for the underlying mechanisms. So, we investigated this compound and mechanisms here. Moreover, SGLT-2 is specifically located in HK-2 cells, so it is reasonable for us to choose this isoform in the cell type. We have not investigated the SGLT-1 except the PCR detection, since SGLT-1 could be ubiquitously expressed in the body, which is not the target for glucose-lowering therapy.

Question 3: The relationship between SGLT1 and SGLT2 and oxidative stress should be clarified.

It is unclear for the relationship between SGLT1 and SGLT2 and oxidative stress, since the tissue distribution of the two isoforms is different. Dapagliflozin is much selective for SGLT-2 and SGLT-2 is highly expressed in proximal tubules, so the contribution of SGLT1 in HK-2 could be weak.

Question 4: Method: how were the doses of dapagliflozin determined in in vivo?

In vivo dosing should be determined by the pharmacokinetics and effectiveness (glucose in blood and urine).

Question 5: The author should add in vivo experiments to further verify the role of dapagliflozin in oxidative stress-induced cell injury in human proximal tubular cells.

The in vivo oxidative stress ($H_2O_2$) induced kidney injury model is rare and not feasible. In addition, it is not realistic for us to do any in vivo animal work during the COVID-19 lockdown period.

Question 6: It is unacceptable that the author made a great deal of discussion that they did not present in the results. The authors have to highlight the novelties of their work.

We have reworded some sentences in the discussion.

6. PLOS authors have the option to publish the peer review history of their article (what does this mean?). If published, this will include your full peer review and any attached files.

Yes, I agree to publish the review history. However, the reviewer’s questions seem to be unrelated to this manuscript.

Do you want your identity to be public for this peer review? For information about this choice, including consent withdrawal, please see our Privacy Policy.
Reviewer #1: No
